# Fishing cat *Prionailurus viverrinus* distribution and habitat suitability in Nepal

**DOI:** 10.1002/ece3.8857

**Published:** 2022-04-23

**Authors:** Rama Mishra, Hans H. de Iongh, Hewig Leirs, Babu Ram Lamichhane, Naresh Subedi, Shekhar S. Kolipaka

**Affiliations:** ^1^ Evolutionary Ecology Group Department of Biology Antwerp University Antwerp Belgium; ^2^ 26660 Wildlife Conservation Association Nepal (WildCAN) Kathmandu Nepal; ^3^ 536319 Leo Foundation Wageningen The Netherlands; ^4^ National Trust for Nature Conservation Lalitpur Nepal

**Keywords:** fishing cat, habitat modeling, MaxEnt, Nepal, protected areas, species distribution

## Abstract

The fishing cat *Prionailurus viverrinus* is a wetland specialist species endemic to South and Southeast Asia. Nepal represents the northern limit of its biogeographic range, but comprehensive information on fishing cat distribution in Nepal is lacking. To assess their distribution, we compiled fishing cat occurrence records (*n* = 154) from Nepal, available in published literature and unpublished data (2009–2020). Bioclimatic and environmental variables associated with their occurrence were used to predict the fishing cat habitat suitability using MaxEnt modeling. Fishing cat habitat suitability was associated with elevation (152–302 m), precipitation of the warmest quarter, i.e., April–June (668–1014 mm), precipitation of the driest month (4–7 mm), and land cover (forest/grassland and wetland). The model predicted an area of 4.4% (6679 km^2^) of Nepal as potential habitat for the fishing cat. About two‐thirds of the predicted potentially suitable habitat lies outside protected areas; however, a large part of the highly suitable habitat (67%) falls within protected areas. The predicted habitat suitability map serves as a reference for future investigation into fishing cat distribution as well as formulating and implementing effective conservation programs in Nepal. Fishing cat conservation initiatives should include habitats inside and outside the protected areas to ensure long‐term survival. We recommend conservation of wetland sites, surveys of fishing cats in the identified potential habitats, and studying their genetic connectivity and population status.

## Graphical abstract

We compiled all available information of fishing cat in Nepal to date and predicted its habitat suitability using MaxEnt modeling. We found a small portion (4.4% of Nepal) as the potential habitat of fishing cat in Nepal. The predicted fishing cat habitat suitability map serves as a reference for future research and formulating effective conservation programs for fishing cats in Nepal.

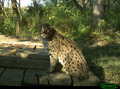

## 1 INTRODUCTION

**FIGURE 1:**
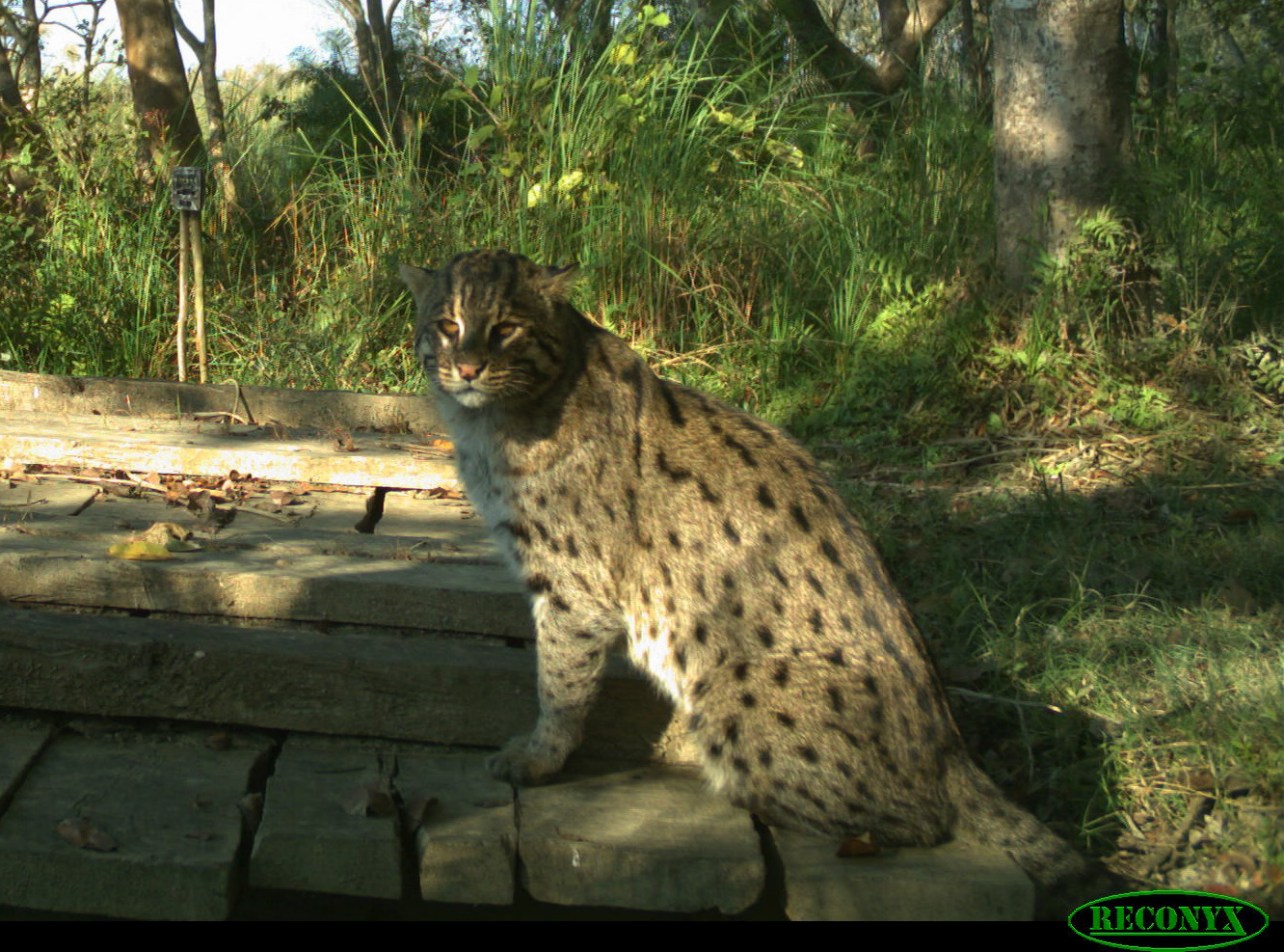
Fishing cat (*Prionailurus viverrinus*) photographed in camera trap in Shuklaphanta National Park, Nepal

The fishing cat (*Prionailurus viverrinus*, Figure 1) is a globally threatened small cat (5–16 kg) categorized as “Vulnerable” in the IUCN Red List (Mukherjee et al., 2016). It is a habitat specialist, strongly associated with wetlands such as swamps, marshes, rivers, and mangroves (Mishra et al., 2018; Mukherjee et al., 2016). Thus, fishing cats have a patchy distribution throughout their range, primarily in lowland areas of South and Southeast Asia (Mishra et al., 2018; Mukherjee et al., 2016; Silva et al., 2020). Their range is decreasing globally with shrinking and degradation of wetlands due to several factors such as the conversion into other land uses, land degradation (increasing erosion and sedimentation), industrialization, urbanization, and global climate change (Chowdhury et al., 2015; Mishra et al., 2020; Mukherjee et al., 2012; Taylor et al., 2016). Fishing cats are also threatened by hunting, retaliatory killing, and road kills (Mishra et al., 2021; Timilsina et al., 2021).

Nepal is one of the fishing cat range countries, with the species distribution believed to encompass large parts of the Southern lowland region called the Terai (Jnawali et al., 2011). However, their actual distribution is not well understood in Nepal. Most of the information on fishing cats is based on opportunistic records during surveys targeted at large charismatic species like tigers *Panthera tigris* (Poudel et al., 2019; Timilsina et al., 2021; Yadav et al., 2018). Fishing cat has been recorded at eight different sites, including five protected areas (Koshi Tappu Wildlife Reserve, Parsa, Chitwan, Bardia, and Shuklaphanta National Parks) and three sites (Sunsari, Bara, and Kapilvastu) outside the protected areas in Nepal's Terai (Mishra et al., 2021). However, the population connectivity among these sites remains unclear. Fishing cat diet broadly includes fish, rodents, birds, amphibians, and other invertebrates (Cutter, 2015; Haque & Vijayan, 1993). Along with the natural habitats, fishing cats have been recorded from agricultural fields and fish farms in all range countries (Chowdhury et al., 2015; Mishra et al., 2021; Mukherjee et al., 2016). Thus, there is the possibility of fishing cat presence in other areas of the Terai. Assessing habitat suitability is necessary to identify the potential sites within the fishing cat range and prioritize for their conservation.

Maximum entropy (MaxEnt) modeling is the most widely used method globally for predicting species distribution and habitat suitability despite its criticism as a machine learning technique (Elith et al., 2011; Guillera‐Arroita et al., 2015). It uses presence‐only data for predicting potential areas of the species occurrence (Phillips et al., 2006). In this study, we compiled fishing cat occurrence records together with bioclimatic and environmental variables and predicted habitat suitability of fishing cat in Nepal using maximum entropy (MaxEnt) models. Our research questions were as follows:

1. Where is fishing cat distributed in Nepal based on photographic evidence?
2. What is the extent of suitable fishing cat habitat in Nepal?
3. Which factors influence the fishing cat habitat suitability?

The predicted suitability indices from this study will guide for fishing cat conservation in Nepal.

## 2 MATERIALS AND METHODS

### 2.1 Study area

The study was carried out across the fishing cat range, i.e., lowland Terai region of Southern Nepal (26˚30’–28˚55’N latitude; 80˚04’–88˚08’E longitude) (Jnawali et al., 2011). The Terai is a highly productive area in the northern part of the Ganga River's floodplain. This region consists of five National Parks (Parsa, Chitwan, Banke, Bardia, and Shuklaphanta), one Wildlife Reserve (Koshi Tappu – KTWR), and a conservation area (Blackbuck) in Nepal. These protected areas (PA) of the Terai support diverse habitats including wetlands. In addition, this region also includes four Ramsar sites (wetlands of global importance), two inside (KTWR and Beeshazar) and two outside (Jagdishpur and Ghodaghodi) of the PAs.

The Terai has a sub‐tropical climate with four distinct seasons: winter (mid‐December/mid‐March), pre‐monsoon (mid‐March/mid‐June), monsoon (mid‐June/mid‐September), and autumn (mid‐September/mid‐December; Lamichhane et al., 2018). May and June are the hottest months where the mean monthly temperature ranges 35–40°C. January is the coolest month, with 14–16°C in mid‐winter. The mean annual rainfall ranges from 1138 to 2680 mm, with over 80% of the rain occurring during the three monsoon months (mid‐June to mid‐September). Until the early 1900s, most of Nepal's Terai was covered by forests, grasslands, wetlands, and rivers, but after the eradication of Malaria in the mid‐1950s, large numbers of people from the hills migrated to the Terai and settled, thereby cutting down large tracts of forests for agriculture and settlements (Lamichhane et al., 2018). At present, about 55% of Nepal's population lives in the Terai (~14% land area) (Central Bureau of Statistics [CBS], 2011).

**FIGURE 2:**
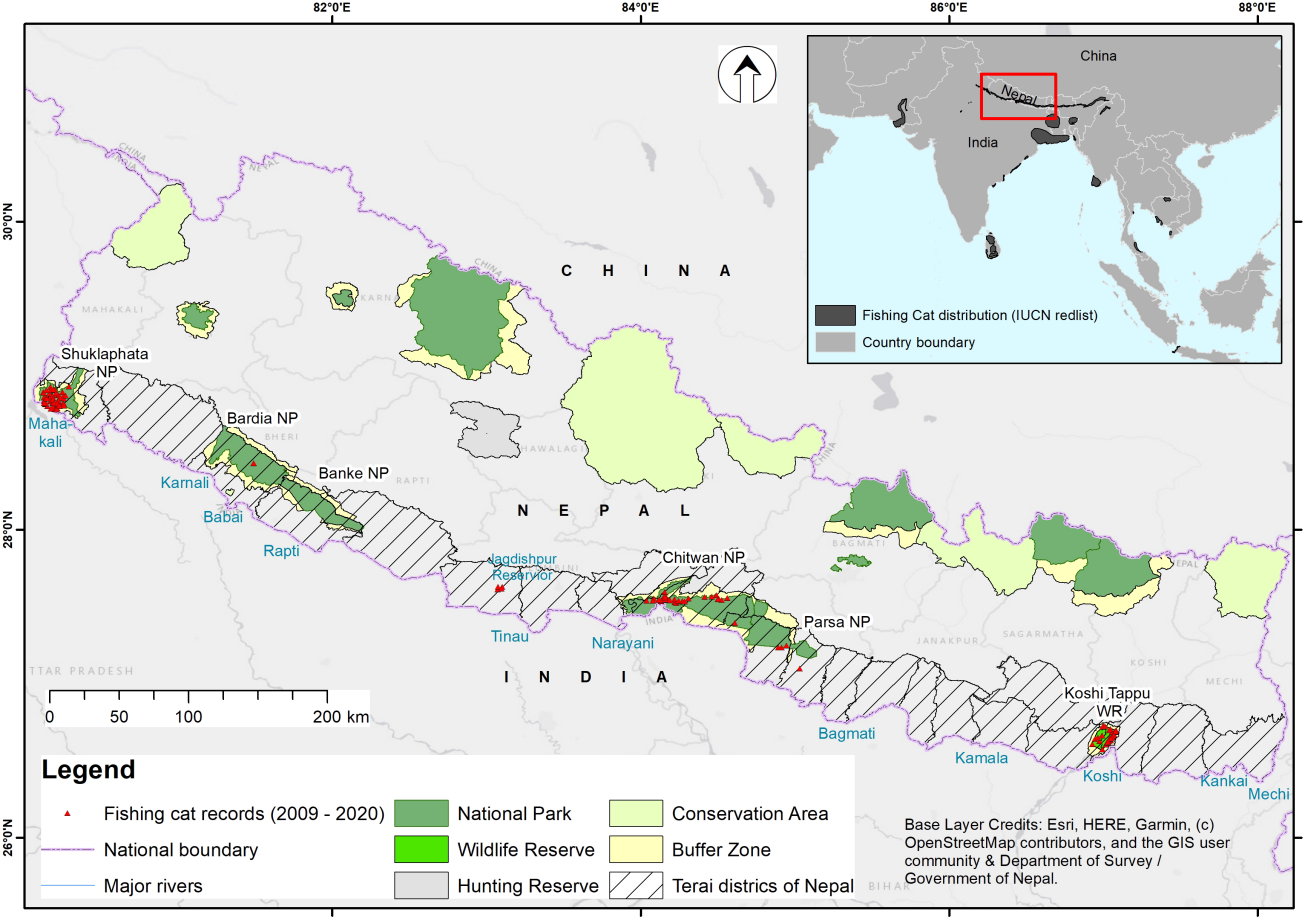
Map of Nepal showing fishing cat records, major rivers, Ramsar sites, and protected areas

Nepal's Terai contains catchments of 10 major river system – Mechi, Kankai, Koshi, Kamala, Bagmati, Narayani, Tinau, West Rapti, Karnali, Babai, and Mahakali (Figure 2), where four of them (Koshi, Narayani, Karnali, and Mahakali) originating from the snow‐covered Himalayas. Passing through the Terai region of Nepal, all the rivers drain into the Ganga River system in India. The Terai PAs also support many oxbow lakes created by these river systems. However, due to siltation and succession, they are gradually converted into marshes or swamps, and ultimately grasslands. These wetlands host high floral diversity (>250 species are exclusively aquatic) and provide breeding, roosting and feeding sites to many migratory and resident wetland birds, and food and habitat for fish and several invertebrates (IUCN Khadka et al., 2017; Kumar et al., 2011; Nepal, 2004). Outside the PAs a large part of the Terai of Nepal is covered by human settlements and farming lands. Rice is the most important crop of Nepal, and the Terai region produces the majority of it (Subedi & Paudel, 2020). Fish farming has been increasing in recent years, and paddy fields with a permanent water source are often converted into fish ponds (Aryal et al., 2020; Husen, 2019; Mishra et al., 2020).

### 2.2 Fishing cat occurrence records

We compiled all the published data and available unpublished records of fishing cats in Nepal between 2009 and 2020, including camera trapping data, live records with photographic evidence, and carcasses of fishing cats (Table 1). We did not include pugmark records due to possible misidentification with other small cats (Palei et al., 2018). The records of fishing cats were compiled, along with the location details and GPS coordinates of each sighting. We also included details of the fishing cat images/videos such as date, time, and habitat type. A total of 312 detections of fishing cats (1 live photo capture, 2 carcasses, and 309 detections in camera traps from 1465 images and 126 videos) were compiled from 154 locations. Of these locations, 118 were from core protected areas, 31 from the buffer zones, and 5 were outside the protected areas (Table 1). Higher number of fishing cat presence locations inside the protected areas were obtained probably because of the high density and more survey efforts there.

**TABLE 1:** Presence data of fishing cats between 2009 and 2020 with number of location (and detections in parenthesis) used in this study and their sources (“NA” means “information not available”). Unpublished data from camera trapping was obtained during tiger surveys in respective sites

SN	Site	Year	Type of evidence	No. of location (No. of detection)	Source
1	Koshi Tappu Wildlife Reserve Buffer Zone	2013	Camera trap	9 (9)	Taylor et al. (2016)
2	Koshi Tappu Wildlife Reserve Buffer Zone	2016	Camera trap	16 (57)	Mishra et al. (2021)
3	Koshi Tappu Wildlife Reserve Buffer Zone	2017	Camera trap	6 (24)	Mishra et al. (2021)
4	Koshi Tappu Wildlife Reserve	2017	Camera trap	12 (52)	Mishra et al. (2021)
5	Bara District (Agricultural area)	2020	Roadkill	1 (1)	Mishra et al. (2021)
6	Parsa National Park	2016	Camera trap	3 (3)	Poudel et al. (2019)
7	Chitwan National Park	2009	Camera trap	9 (NA)	DNPWC unpublished data
8	Chitwan National Park	2010	Camera trap	3 (4)	Karki (2011) and DNPWC (2010)
9	Chitwan National Park	2011	Carcass record	1 (1)	Chitwan National Park Office
10	Chitwan National Park	2012	Camera trap	4 (7)	Mishra (2013)
11	Chitwan National Park	2013	Camera trap	5 (7)	Mishra (2013)
12	Chitwan National Park	2016	Photographic record	1 (1)	Mishra et al. (2018)
13	Chitwan National Park	2018	Camera trap	8 (12)	Amy Fitzmaurice & DNPWC unpublished data
14	Kapilvastu District (Jagdishpur reservoir)	2014	Camera trap	4 (NA)	Dahal et al. (2014)
15	Bardia National Park	2017	Camera trap	1 (2)	Yadav et al. (2018)
16	Shuklaphanta National Park	2013	Camera trap	11 (14)	DNPWC/ShNP/NTNC unpublished data
17	Shuklaphanta National Park	2014	Camera trap	5 (11)	DNPWC/ShNP/NTNC unpublished data
18	Shuklaphanta National Park	2015	Camera trap	10 (27)	DNPWC/ShNP/NTNC unpublished data
19	Shuklaphanta National Park	2016	Camera trap	10 (16)	Yadav et al. (2020)
20	Shuklaphanta National Park	2017	Camera trap	18 (26)	DNPWC/ShNP/NTNC unpublished data
21	Shuklaphanta National Park	2018	Camera trap	17 (25)	DNPWC/ShNP/NTNC unpublished data
Total locations				154 (312)	

#### 2.2.1 Spatial filtering of data

Spatial filtering of the fishing cats’ recorded locations was carried out at the scale of 6.25 km^2^ (grid cell of 2.5 × 2.5) based on the home range of a female fishing cat (4–8 km^2^; Mishra et al., 2018; Sunquist & Sunquist, 2002). Single locations within the grid cells were extracted randomly and used in MaxEnt modeling (Kramer‐Schadt et al., 2013). Of 154 locations of fishing cats, 79 remained after spatial filtering, including 64 in core areas of national parks and wildlife reserve.

### 2.3 Environmental variables

We used 21 environmental variables including elevation and land cover and 19 bioclimatic variables (Table 2) to construct the MaxEnt model. The bioclimatic variables are important predictors of environmental niche of a species. We selected elevation and land cover based on habitat specialist nature of fishing cats and their distribution in lowland areas only. Bioclimatic variables at approximately 1 × 1 km^2^ spatial resolution were obtained from WorldClim – Global Climate Data (http://www.worldclim.org/) – of 1950 to 2000 (Hijmans et al., 2005). In addition, elevation and land cover data were obtained from SRTM DEM (90 m resolution) and global land cover data from DIVA‐GIS, respectively (Robinson et al., 2014). The land cover included 22 different types, including forests/grasslands, wetlands, agriculture, and settlement/built up area.

**TABLE 2:** Environmental variables used in the model development (bold variables used in the MaxEnt analysis)

Code	Environmental variables	Unit
**bio1**	**Annual Mean Temperature**	**°C**
**bio2**	**Mean Diurnal Range (Mean of monthly max temp – min temp)**	**°C**
bio3	Isothermality (bio 2/bio 7) (*100)	%
bio4	Temperature Seasonality (standard deviation *100)	°C
bio5	Max Temperature of Warmest Month	°C
**bio6**	**Min Temperature of Coldest Month**	°C
bio7	Temperature Annual Range (bio5–bio6)	°C
**bio8**	**Mean Temperature of Wettest Quarter**	°C
bio9	Mean Temperature of Driest Quarter	°C
bio10	Mean Temperature of Warmest Quarter	°C
bio11	Mean Temperature of Coldest Quarter	°C
bio12	Annual Precipitation	mm
bio13	Precipitation of Wettest Month	mm
**bio14**	**Precipitation of Driest Month**	**mm**
bio15	Precipitation Seasonality (Coefficient of Variation)	numeric
bio16	Precipitation of Wettest Quarter	mm
bio17	Precipitation of Driest Quarter	mm
**bio18**	**Precipitation of Warmest Quarter**	**mm**
**bio19**	**Precipitation of Coldest Quarter**	**mm**
**elev**	**Elevation**	**m**
**lcover**	**Land cover**	**Categories**

All variables were converted into ASCII format as required for the modeling in MaxEnt and clipped by the boundary of Nepal in ArcGIS 10.4. First, we extracted each environmental variable corresponding to each fishing cat occurrence and ran a correlation test in SPSS 22 (Xu et al., 2019). Then, the Pearson correlation coefficient (*r*) was calculated and one of each pair of highly correlated (*r* > 0.7) variables were eliminated (Dormann et al., 2013) to improve the accuracy of the model simulation further. While eliminating the variables, we kept those more relevant to explain fishing cat distribution. This resulted in nine variables for further MaxEnt analysis (variables in bold in Table 2).

### 2.4 Distribution modeling

We used maximum entropy model in MaxEnt software 3.4.1 (Phillips et al., 2006) to predict the fishing cats’ potential habitat. MaxEnt is a widely used species distribution model (SDM) for predicting species distribution when species records are available in the form of presence‐only data (Elith et al., 2011; Xu et al., 2019). This model provides the probability of occurrence of a given species, ranging from 0 to 1, and the closer the value is to 1, the greater the probability of species occurrence (Phillips et al., 2006). Although there are critics about the reliability of habitat suitability predictions from presence‐only data (Guillera‐Arroita et al., 2015), we used this method due to the lack of true absence data.

All the fishing cat occurrence records and the nine selected environmental variables were loaded into the MaxEnt software to run the model. Jackknife tests were performed in MaxEnt to know the weight of each variable. A total of 25% of the recorded distribution data were randomly selected as the test set and the remaining as the training set. The algorithm ran for either 1000 iterations of these processes or continued until the convergence threshold equaled 0.00001. The area under the receiver operating characteristic (ROC) curves (AUC) was utilized to assess the model performance. The AUC is an effective threshold‐independent index to discriminate presence from absence (or background locations generated automatically) (Liu et al., 2013; Xu et al., 2019). The threshold value based on the AUC of the ROC ranges from 0 to 1. An AUC score of 1 indicates perfect prediction, whereas a value of 0.5 indicates random prediction. The accuracy of model simulation results is related to the AUC value. AUC values >0.9 are considered as excellent, 0.8 < AUC < 0.9 as good, 0.7 < AUC < 0.8 as fair, and AUC < 0.7 as a poor prediction (Araújo et al., 2005). The model predicts the value of fishing cat habitat suitability for each pixel (~1 km^2^) between 0 and 1, where 0 means not suitable and 1 is perfectly suitable. The predicted habitat was categorized into four categories; no suitability (0–0.2), low suitability (0.2–0.4), medium suitability (0.4–0.6), and high suitability (0.6–1) (Xu et al., 2019).

## 3 RESULTS

### 3.1 Fishing cat distribution records

The fishing cat locations range between 26.57° and 28.91°N Latitudes; 80.14° and 87.08°E longitudes; and 81 and 307 m altitude in Nepal's Terai (Figure 2). The highest number of records (*n* = 71) were obtained from Shuklaphanta NP followed by Koshi Tappu WR (*n* = 43). Based on the records, fishing cats show a discontinuous distribution in Nepal with five distinct clusters, i.e., Koshi Tappu WR in the east, Chitwan NP‐Parsa NP‐Bara district complex in central Nepal, Jagdishpur area in the west, Bardia NP in mid‐western, and Shuklaphanta NP in the far west (Figure 2).

### 3.2 Key environmental variables contributing to fishing cat distribution

**FIGURE 3:**
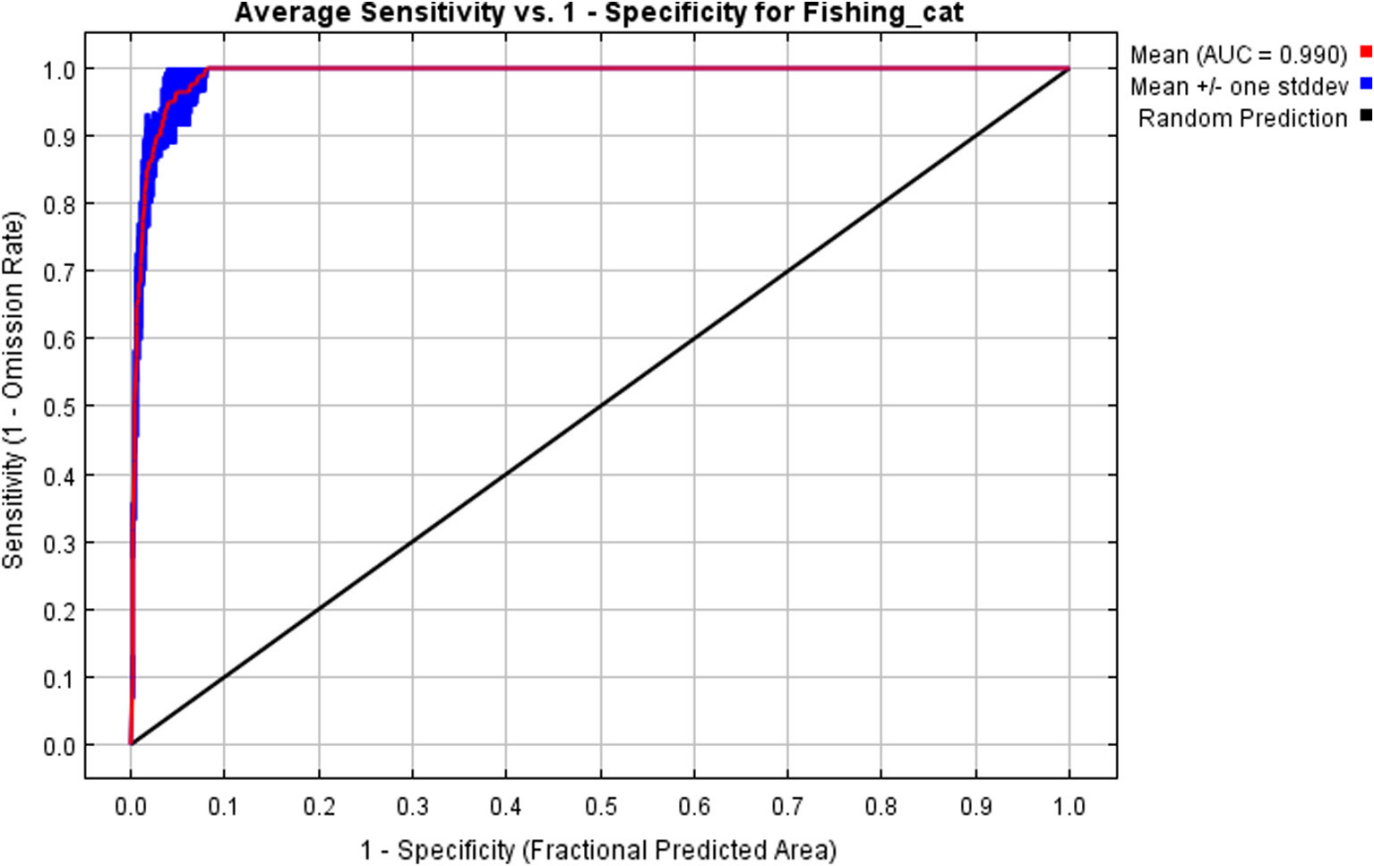
Area under the receiver operating characteristic curve (AUC) of MaxEnt prediction. The high value (closer to 1) represents better prediction

**FIGURE 4:**
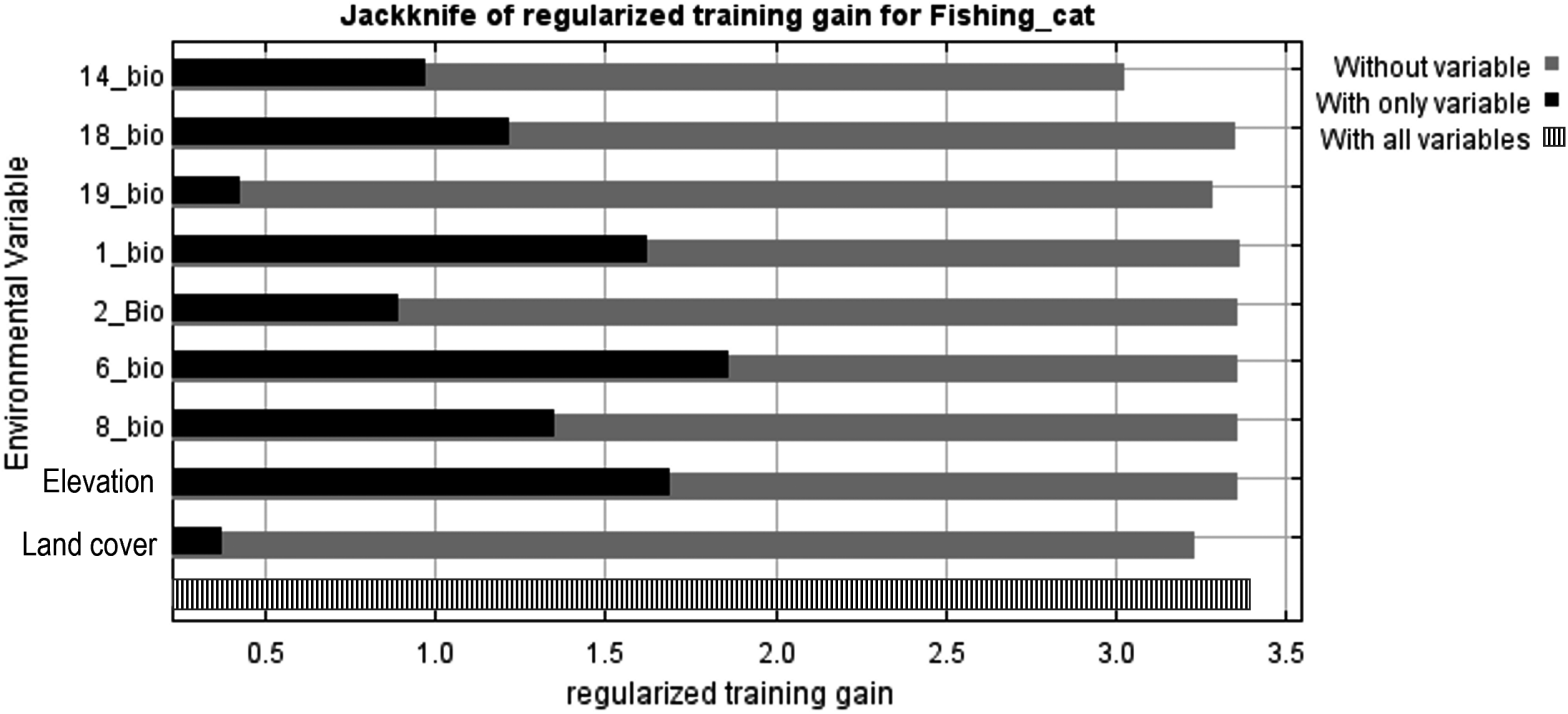
Importance of environment variables to fishing cat by Jackknife analysis, where 14_bio = Precipitation of Driest Month, 18_bio = Precipitation of Warmest Quarter, 19_bio = Precipitation of Coldest Quarter, 1_bio = Annual Mean Temperature, 2_boi = Mean Diurnal Range (Mean of monthly max temp – min temp), 6_bio = Min Temperature of Coldest Month, and 8_bio = Mean Temperature of Wettest Quarter. (Black bar = importance of each variable in explaining the variation in the data when use separately. Grey bar = loss in total model gain when the particular variable was omitted, indicating the presence of unique information necessary for explaining the model. Bar with lines pattern = total model gain)

The ROC of the constructed MaxEnt model (Figure 3) showed the AUC value of 0.990, indicating excellent performance of the model. The results of the jackknife test provided the contributions of each key environmental variable to the model (Figure 4). Of 21 covariates selected in distribution modeling, 9 had high influence in determining the distribution of fishing cats (Table 3) including elevation, land cover, and seven bioclimatic variables (Bio 1, 2, 6, 8, 14, 18, and 19). Collectively, these nine variables contributed for 99% of the variation in model. Four variables were responsible for predicting >5% of the variation in the data (Table 3). Elevation was the most important variable with 32.3% contribution, followed by precipitation of the warmest quarter (18_bio), precipitation of the driest month (14_bio), and land cover. Five variables: mean temperature of wettest quarter (8_bio); annual mean temperature (1_bio); precipitation of coldest quarter (19_bio); minimum temperature of coldest month (6_bio); and mean diurnal range (2_bio) contribute the least (each below 5%) to the distribution of the species (Table 3).

**TABLE 3:** Relative contribution of environmental variables to the MaxEnt model used to map the potential habitat of the fishing cat in Nepal

Variables	Percent contribution	Permutation importance
Elevation	32.3	3
Precipitation of Warmest Quarter	22.8	4.7
Precipitation of Driest Month	19.7	15.9
Land cover	9.1	1.5
Mean Diurnal Range (mean of monthly (max temp–min temp))	4.9	6.2
Min Temperature of Coldest Month	4.2	1.6
Precipitation of Coldest Quarter	3.4	4.7
Annual Mean Temperature	2.3	62.4
Mean Temperature of Wettest Quarter	1.3	0

The results indicated that the highest regularized training gain (1.8) for the fishing cat in the present model occurred when the minimum temperature of the coldest month (6_bio) was used in isolation for running the model (Figure 4). This variable is followed in importance by elevation; annual mean temperature (1_bio); mean temperature of the wettest quarter (8_bio); and precipitation of the warmest quarter (18_bio) with the regularized training gain >1.0. The order of importance of the remaining key environmental factors is the precipitation of the driest month (14_bio); mean diurnal range (mean of monthly (maximum temperature – minimum temperature)) (2_bio); precipitation of coldest quarter (19_bio); and land cover. The training sample gain was the lowest after omitting the precipitation of the driest month (14_bio) from the model, suggesting its crucial role in identifying suitable habitat for the fishing cat (Figure 4).

### 3.3 Predicted habitat suitability

**FIGURE 5:**
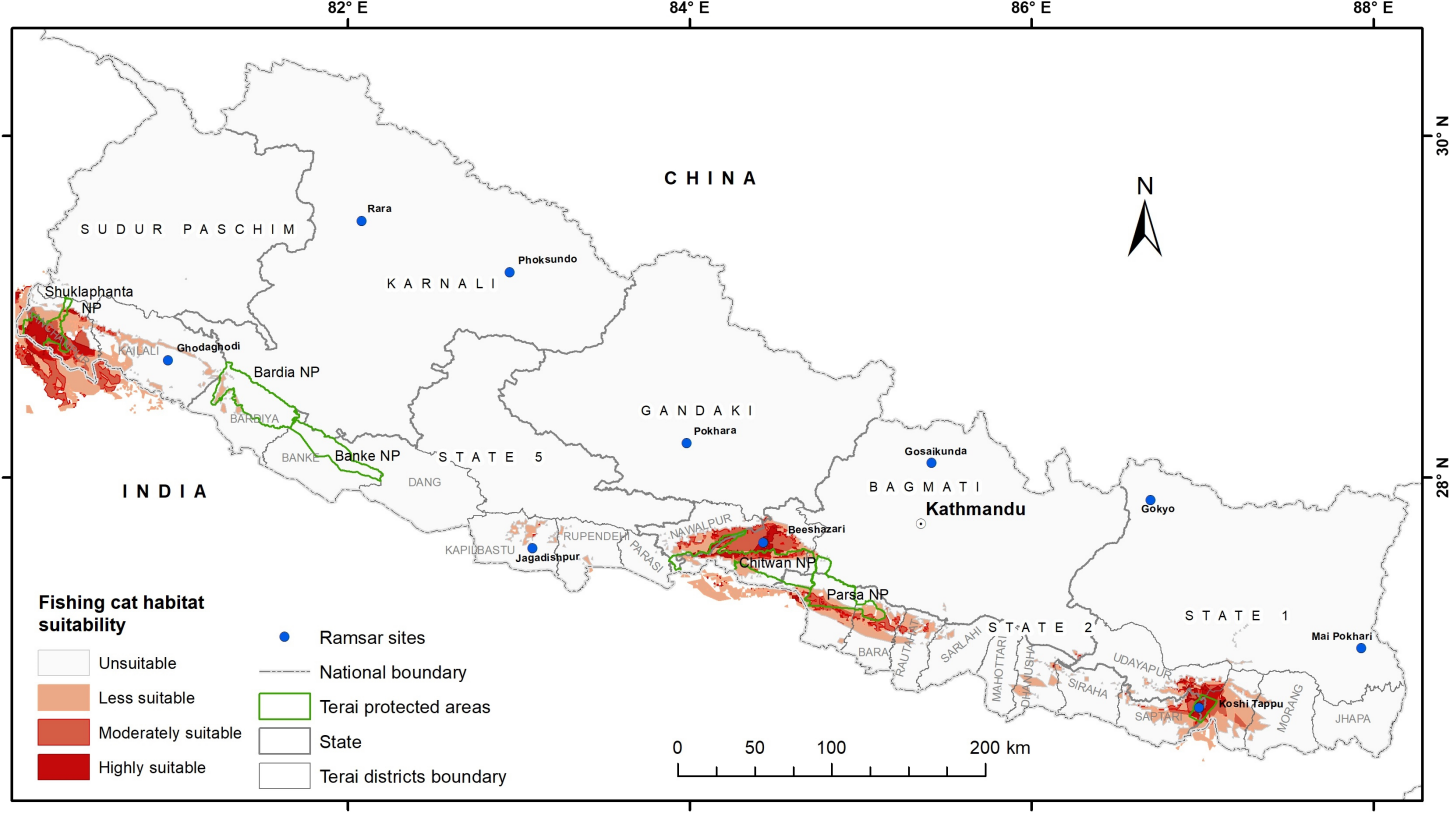
Map of fishing cat (*Prionailurus viverrinus*) habitat suitability in Nepal's Terai and Chure region (NP = National Park)

The model predicted a total of 6679 km^2^ of Nepal as potential habitat for fishing cats (Figure 5). Of this, a 992 km^2^ area was predicted as highly suitable, 1881 km^2^ as moderately suitable, and 3806 km^2^ as less suitable (Table 4). A large part of Nepal (141,733 km^2^) was predicted as unsuitable for the fishing cat. The suitable range of environmental variables for fishing cat distribution based on response curves (Appendix S1) in the MaxEnt model was calculated and presented in Table 5.

**TABLE 4:** Habitat suitability for fishing cat based on model developed in MaxEnt. Only 4.4% of Nepal is predicted as potential fishing cat habitat (cells with values >0.2 in predicted distribution) and 95.6% (141,732.8 km^2^) is unsuitable (<0.2)

Habitat suitability	Area (km^2^)			Area (km^2^)		
	Eastern	Central	Western	Inside PA (%)	Outside PA (%)	Total
Less suitable (0.2–0.4)	1257.5	1409.9	1138.4	942.8 (26)	2863.0 (75)	3805.8
Moderate suitable (0.4–0.6)	408.4	1028.0	444.6	736.9 (39)	1144.1 (61)	1880.9
Highly suitable (>0.6)	261.9	358.7	371.8	664.2 (67)	328.3 (33)	992.4
Total	1927.8	2796.6	1954.7	2343.9 (35)	4335.40 (65)	6679.1

**TABLE 5:** Suitable range of environmental variables for potential distribution of fishing cat

Environmental variables/unit	Suitable range	Optimum value
Annual Mean Temperature/°C	23.81–24.66	24.37
Mean Diurnal Range (Mean of monthly (max temp–min temp))/°C	11.14–12.18	11.80
Min Temperature of Coldest Month/°C	9.03–10.29	9.85
Mean Temperature of Wettest Quarter/°C	27.82–28.84	28.56
Precipitation of Driest Month/mm	4.27–6.98	5.53
Precipitation of Warmest Quarter/mm	668.12–1014.35	721.19
Precipitation of Coldest Quarter/mm	18.08–81.07	23.56
Elevation/m	152.08–302.72	202.29
Land cover^a^	Wetlands (0.79), Forest/grasslands (0.75)	Wetlands

^a^ Categorical variable (22 categories); only two categories “Wetlands” and “Forest & grassland” had predicted value above 0.4 (i.e., suitable habitat).

## 4 DISCUSSION

This is the first comprehensive study of fishing cat distribution and prediction of their potential habitat in Nepal. Only 4.4% of Nepal was found to be potential fishing cat habitat, mostly in the lowland Terai (<300 m). Despite a few fishing cat presence locations, the majority (65%) of the potentially suitable habitat lies outside the PAs. However, two‐thirds of the “highly suitable” habitat lies within PAs (Figure 5). The fishing cat population within the PAs receives the highest level of protection. Among the protected areas, the highest number of fishing cat records were obtained from Shuklaphanta National Park, followed by Koshi Tappu Wildlife Reserve, indicating a high density of fishing cats in those sites (Mishra et al., 2021). In contrast, there were only a few records outside the PAs indicating less survey effort or their low density (Table 1).

The MaxEnt model of our study had high discriminative ability (AUC = 0.990), suggesting it is reliable for defining suitable areas for fishing cats in Nepal. The geographic range of the fishing cat is relatively small in Nepal and we had compiled all available locations of fishing cat presence, representing diverse habitats, allowing us to predict potential habitat. Moreover, our target species is a habitat specialist, which likely contributed to high discriminative ability. To reduce the sampling bias and a clustering effect from multiple locations in a single site, we also carried out spatial filtering based on the home range size of fishing cats (Boria et al., 2014). Thus, we believe the habitat suitability predicted by the model well represents the fishing cat range in Nepal. A recent (2021) camera trapping survey conducted by the first author's team in Chitwan and Shuklaphanta National Parks detected fishing cats in the locations predicted as suitable habitat, indicating the reliability of the model.

However, the result of MaxEnt model based on presence‐only records has its limitations. The presence records of the fishing cat were predominantly from the PAs. A very small number of locations outside protected areas was a constraint of our study which may have resulted in the sampling bias resulting large extent of highly suitable habitat inside PAs. Thus, we suggest carrying out the study on occupancy framework accounting for the detection probability covering the entire fishing cat habitat including the non‐protected areas.

Our results show that elevation, precipitation of driest months, precipitation of the warmest quarter, and land cover are the most important variables predicting the fishing cat habitat suitability. Furthermore, the jackknife test of variable importance shows that the environmental variable with the highest gain when used in isolation is the “Min Temperature of the Coldest Month.” The environmental variable that decreases the gain the most when it is omitted (have the most information that is not present in the other variables) is the “Precipitation of the Driest Month.”

We found fishing cats below 310 m in the lowland Terai of Nepal, and similar records are reported throughout the range. However, in Sri Lanka, the fishing cat has been recorded up to 1800 m altitude (Mukherjee et al., 2016). Precipitation in the driest month and the wettest quarter had high contributions for predicting the fishing cat distribution. Similar findings on the influence of precipitation of the driest month have been reported in Bangladesh (Rahman, 2017). It indicates that precipitation is important for the higher suitability of fishing cat habitat as it can temporarily expand the wetland area during flooding.

The narrow suitable range of these predictor variables indicates the narrow environmental niche of fishing cats. Therefore, alteration in temperature and precipitation will potentially affect fishing cats’ niche. Amid global climate change, the annual average, average minimum, and average maximum temperature of Nepal's Terai are increasing. In contrast, the annual average precipitation is decreasing, with decreases in both pre‐monsoon and monsoon precipitation in recent years (Thapa & Dhulikhel, 2019). Such trend is expected to continue, which could reduce the suitable range of fishing cats in future.

The model showed the vital role of wetlands, and forest and grassland cover for the occurrence of fishing cats (Mishra et al., 2018; Palei et al., 2018). However, the freshwater wetland ecosystem is vulnerable to various threats such as shrinkage, decreasing water volume, the spread of invasive species, physical/chemical pollution, and climate change (Chaudhary et al., 2016; Lamsal et al., 2017). Wetland surveys in Chitwan documented drying of some wetlands (converted into grasslands or forests) and one‐quarter of them were in bad condition (Khadka et al., 2015), affecting fishing cat distribution. The first author failed to obtain fishing cats in camera traps (2012) from a location in the eastern sector of Chitwan NP where four fishing cat individuals were radio‐collared during the 1980s (Mishra, 2013; Mishra et al., 2021; Sunquist & Sunquist, 2002). The marshes at this location in the 1980s have since completely dried and converted into grasslands. This is an example of the rapid habitat conversion threatening wetland specialist species like fishing cats.

The potential habitat predicted by the model shows three population clusters (Eastern – Koshi to Dhanusa, Central – Sarlahi to Kapilvastu, and Western – Dang to Kanchanpur) with possible connectivity between the eastern and central populations. In addition, the model predicted that most of fishing cat habitat occurs outside the PAs. However, there are only little efforts to understand fishing cat status in those areas. Therefore, we recommend conducting a broader fishing cat survey in the potential habitats identified by the model where information is limited such as Saptari, Dhanusa, Rautahat, Bara, Chitwan (Madi area), Kapilvastu, and Kailali districts. Fishing cats using the human‐dominated areas indicate that they are relatively tolerant of human disturbance. Similar findings of fishing cat presence outside PAs are reported in neighboring India, where half of the fishing cat populations are believed to occur in unprotected habitat (Kolipaka et al., 2019). In Thailand, Cutter (2015) reported extensive use of paddy fields by fishing cats. In Sri Lanka, fishing cats also occur in peri‐urban areas of Colombo (Ratnayaka, 2021). In Nepal Terai, fisheries are expanding at the expense of agricultural areas, creating both opportunities (additional wetland habitats with abundant fish) and challenges (risks of retaliatory killing in the fish ponds) (Mishra et al., 2021).

## 5 CONSERVATION IMPLICATIONS AND CONCLUSION

This study has comprehensively presented fishing cat records in Nepal from recent decade, and predicted habitat suitability through maximum entropy model. Our finding is important for conservation planning and prioritization of areas for fishing cat conservation in Nepal. Only a small portion of Nepal's Terai is predicted as potential fishing cat habitat. The majority of predicted fishing cat habitat lie outside the protected areas where they face various threats for survival, including habitat conversion (into agriculture, settlement, and other infrastructure development), persecution, and poaching. In addition, wetland shrinkage and habitat conversion caused by the changing global climate threaten this habitat specialist. Therefore, we recommend investigation of the status of fishing cats in Nepal and possible connectivity among population clusters of this species, and conservation awareness for local stakeholders and communities in predicted potential sites.

## AUTHOR CONTRIBUTIONS

**Rama Mishra:** Conceptualization (equal); Data curation (lead); Formal analysis (equal); Methodology (equal); Project administration (equal); Software (equal); Validation (equal); Visualization (equal); Writing – original draft (lead); Writing – review & editing (equal). **Hans H. de Iongh:** Conceptualization (equal); Project administration (equal); Supervision (lead); Writing – review & editing (equal). **Herwig Leirs:** Conceptualization (equal); Writing – review & editing (equal). **Babu Ram Lamichhane:** Conceptualization (equal); Formal analysis (equal); Software (equal); Validation (equal); Writing – review & editing (equal). **Naresh Subedi:** Conceptualization (equal); Supervision (equal); Writing – review & editing (equal). **Shekhar S. Kolipaka:** Writing – review & editing (equal).

## Supporting information

Appendix S1

## Data Availability

Fishing cat locations, bioclimatic and environmental variables used in this article are available via Dryad (https://doi.org/10.5061/dryad.37pvmcvn5).
