# Supplementary material for: Fishing cat Prionailurus viverrinus distribution and habitat suitability in Nepal
Source: Ecol Evol. 2022 Apr 23;12(4):e8857. doi: 10.1002/ece3.8857 (PMC9034449; doi:10.1002/ece3.8857)
Supplement: Supplementary file 1 — Appendix S1 [file ECE3-12-e8857-s001.docx]

*Appendix 1.* Response curves of environmental variables to distribution probability of fishing cat. The values in x-axis represents the environmental variables and y-axis values represents the predicted probability of suitable conditions. The red line represents the mean response of the multiple replicate runs and the mean ± standard deviation (blue area and two shades for categorical variables).


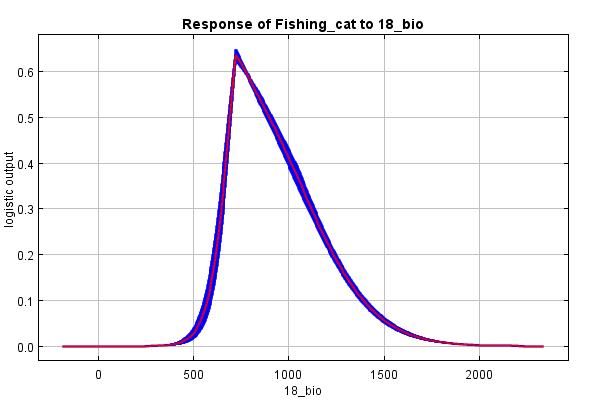


*A1(a). Response of fishing cat to ‘Precipitation (mm) of Warmest Quarter’.*


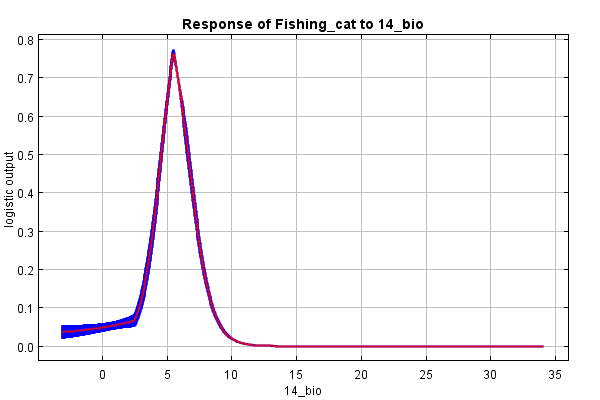


*A1(b). Response of fishing cat to ‘Precipitation (mm) of Driest Month’.*


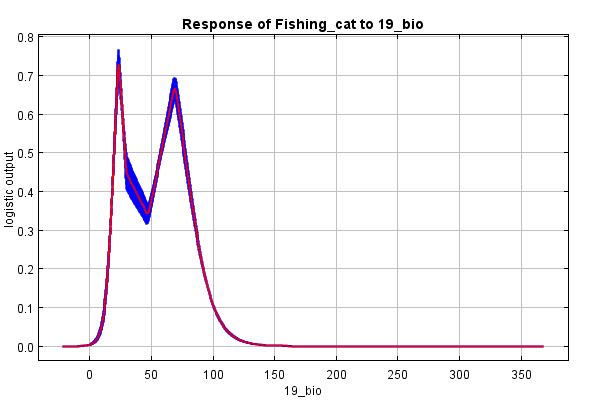


*A1(c). Response of fishing cat to ‘Precipitation (mm) of Coldest Quarter’.*


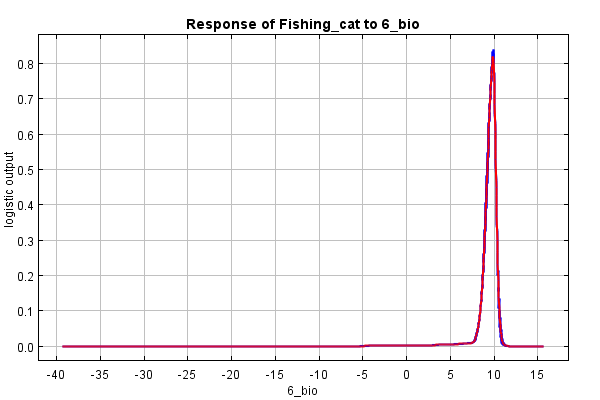


*A1(d). Response of fishing cat to ‘Minimum Temperature (°C) of Coldest Month’.*


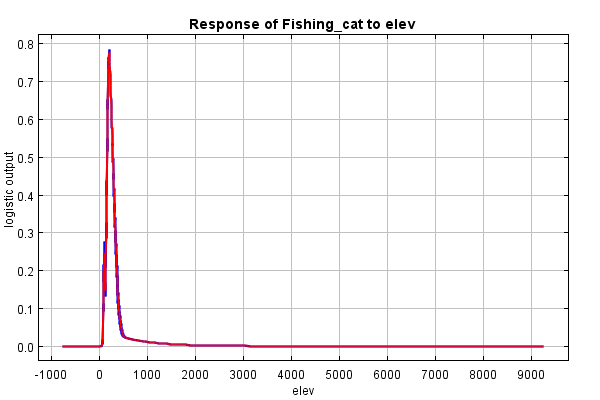


*A1(e). Response of fishing cat to ‘Elevation (m)’.*


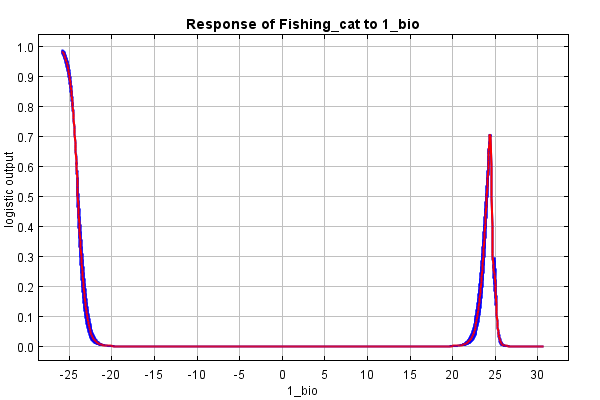


*A1(f). Response of fishing cat to ‘Annual Mean Temperature (°C)’.*


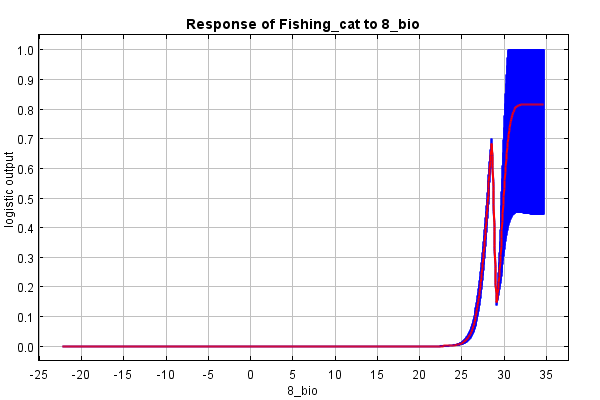


*A1(g). Response of fishing cat to ‘Mean Temperature (°C) of Wettest Quarter’.*


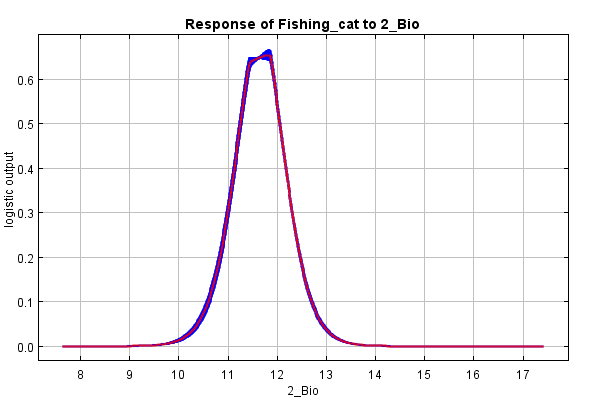


*A1(h). Response of fishing cat to ‘Mean Diurnal Range (Mean of monthly (max temp – min temp)’.*


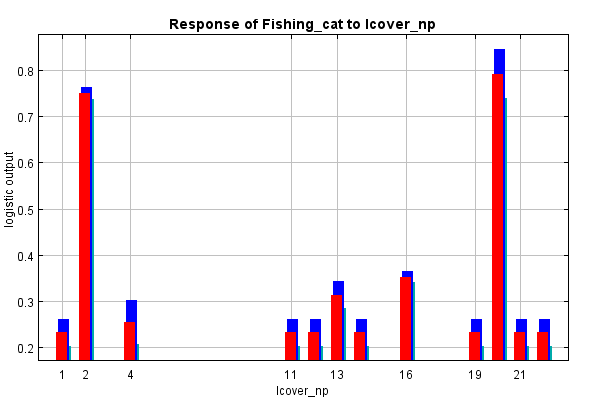


*A1(i). Response of fishing cat to ‘Land cover’. Here the variable ‘Land cover’ is categories in 22 types (in x-axis), where 2 and 20 having high influence in fishing cat distribution are ‘Forest and grassland cover’ and ‘wetlands cover’ respectively. Other categories are 1 – mosaic of cropland and trees, 4 – bare areas, 11 – Broadleaved deciduous closed forest, 12 – Needleleaved evergreen forest, 13 – needleleaved deciduous forest, 14 – open trees, 16 – broadleaved deciduous thicket, 19 – Shrubs on flooded areas, 21 – Builtup areas, 22 – Ice/snow.*
